# Loneliness and Social Distancing During the COVID-19 Pandemic: Risk Factors and Associations With Psychopathology

**DOI:** 10.3389/fpsyt.2020.589127

**Published:** 2020-11-20

**Authors:** Asle Hoffart, Sverre Urnes Johnson, Omid V. Ebrahimi

**Affiliations:** ^1^Modum Bad Psychiatric Hospital, Vikersund, Norway; ^2^Department of Psychology, University of Oslo, Oslo, Norway

**Keywords:** loneliness, risk factors, resilience factors, depression, anxiety, COVID-19, social distancing measures

## Abstract

**Background:** The mitigation strategies employed against the COVID-19 pandemic have severe mental health consequences. In particular, as a result of the social distancing protocols, loneliness is likely to increase. This study investigates (a) potential risk and resilience factors for loneliness in the Norwegian population during the strict social distancing non-pharmacological interventions (NPIs) implemented against the pandemic and (b) the associations between loneliness and psychopathology symptoms.

**Methods:** A survey was disseminated online to the adult Norwegian population when strict social distancing measures had been implemented for 2 weeks. The resulting sample of 10,061 respondents was unproportionate in terms of gender and educational level and thus sensitivity analyses were conducted. The levels of loneliness were compared across demographic sub-groups, and regression analyses were conducted to identify potential risk and resilience factors for loneliness and associations between loneliness and psychopathology symptoms.

**Results:** Among the stable factors, being single and having a psychiatric diagnosis were related to more loneliness with small effect sizes. Among the state risk factors, more rumination and worry in general were associated with stronger loneliness, showing a medium effect size. Among the coping behaviors examined, doing new things at home not done otherwise was negatively related to loneliness, with a small effect size. Loneliness was associated with both depression and anxiety with small effect sizes when all potential confounders and psychiatric diagnosis were controlled for. The relationship to depression was more marked than the relationship to anxiety.

**Conclusions:** The findings suggest that singles and those with a psychiatric diagnosis were most affected by loneliness during the implementation of social distancing measures to impede the coronavirus. The results support the link between loneliness and depression and anxiety symptoms. The results of the analysis of potential risk and resilience factors point to intervention targets for reducing loneliness during pandemic crises.

## Introduction

The coronavirus disease 2019 (COVID-19) is now a full-blown pandemic with strong effects on global public health (1). While awaiting the development of an effective vaccine, many countries have implemented non-pharmacological interventions (NPIs) involving a variety of social distancing measures to limit the spread of the virus (1).

The pandemic and social distancing protocols used to impede the virus have severe mental health consequences (2). In particular, loneliness is likely to increase, as has been documented during previous pandemics (3). Loneliness can be defined as an unpleasant state of sensing a discrepancy between the desired amount and quality of social interaction and that which is available from the person's environment (4). This definition underscores the fact that feeling alone or lonely depends on one's personal standards for a satisfying social connection and thus does not necessarily mean being alone nor does being alone necessarily mean feeling alone. Thus, social isolation and loneliness are different concepts. However, the social distancing measures restrict the availability of social contact by isolating individuals and families in their homes and separating them from colleagues, friends and relatives. Thus, the NPIs may intensify pre-existing loneliness and elicit detrimental levels of loneliness in individuals not particularly affected by this problem before the NPIs were implemented (2). Loneliness is characterized by a painful state with large neurological similarities to physical pain, with findings revealing similar somatosensory representations in the brain between physical pain and social rejection (5). Loneliness is associated with suicidal ideation and parasuicidal behavior (6) and contributes to a range of somatic (e.g., cardiovascular diseases, hypertension) and mental (e.g., depression, anxiety) conditions (7). Thus, loneliness is linked to overall morbidity and mortality (8). Both objective social isolation and subjective loneliness have been found to increase mortality by about 30% (8), and recent reports indicate that loneliness is now the most lethal problem in Great Britain (9). Consequently, increased loneliness due to the social distancing measures represents a serious mental health problem, and it may contribute in particular to increased depression and anxiety. Moreover, NPI-related loneliness, depression and anxiety may continue even after the social distancing measures are lifted because of self-sustaining feedback between the symptoms of these states (10). For instance, it is known that triggering events can cause loneliness and depression long after the triggers have disappeared (11). Indeed, studies from previous pandemics have indicated the long-term impacts of mitigation strategies such as quarantine on mental health and behavior (12).

Many stable risk factors for experiencing loneliness during the NPIs are those found for experiencing loneliness in general. With respect to age, studies have indicated that loneliness decreases across the life span (13). Other studies have evidenced a more complex trajectory, showing elevated levels among the youngest and oldest adults (14). Lower socioeconomic status, which is closely related to educational level, not actively working and being single have all been found to increase the risk of loneliness (15). Adults with mental disorders have also proved to have increased loneliness (16). For other stable factors, the evidence base is more uncertain. One meta-analysis showed gender to be unrelated to loneliness (17). However, other studies have found loneliness to be stronger among women (18). Not having children has also been proposed as a risk factor, but this hypothesis has received mixed support (18, 19). Being a refugee in a new foreign country could also lead to loneliness, but this situation has been less studied. Thus, among the stable factors, being younger, single, less educated, unemployed and having a psychiatric diagnosis have all been supported as risk factors for loneliness, whereas the evidence for being female, childless and a refugee is mixed or lacking.

Loneliness is also likely to be influenced by specific state variables related to the pandemic and NPIs. The pandemic will naturally elicit more worry and anxiety about health (20). Moreover, NPIs have many work-related and economic consequences such as the shutdown of enterprises and factories and the laying off and dismissal of employees, leading to widespread uncertainty about one's private economy and job. Thus, the pandemic and the NPIs involve many losses and uncertainties with previous pandemics revealing increases in rumination and worry (20). These perseverative thinking activities are likely to increase loneliness in several ways. First, incessant rumination, worry and reassurance-seeking often become frustrating for others and may result in considerable strain within the family, thus, increasing feelings of loneliness (21). Second, rumination and worry take up time and promote an inward focus that interferes with attention to other people and leads to loss of contact with others and reduced social activity (22). Third, rumination typically leads to views that the situation is hopeless and that nothing can be done to overcome it (21). For instance, individuals brooding about their loneliness may lead to the conclusion that seeking contact will fail. Worry increases appraisals of the probability and cost of negative outcomes of future events (e.g., “They will reject me,”) (23). Both of these internal activities may provide reasons for behavioral withdrawal, leading to less access to experiences that could disconfirm the very thoughts strengthened by rumination and worry. The results of these processes are likely to be increased feelings of loneliness, depression, and anxiety. In sum, rumination and worry may promote loneliness through several mechanisms, including strain in close relationships, loss of contact with others, and behavioral inactivity that hinders access to corrective experiences. Depression and anxiety may be parallel effects of these mechanisms (24), but they may also more directly affect and be affected by loneliness (21). Empirically, associations between rumination and reduced social support have been found among bereaved adults (25) and between rumination and loneliness among students (26). Consequently, among state variables affected by the pandemic and the NPIs, rumination and worry–both in general and specifically related to uncertainty about health, work and economy–are likely to amplify loneliness.

However, loneliness may be mitigated by behaviors designed to cope with the home-based isolation situation following implementation of the NPIs. The present pandemic situation provides individuals with opportunities to do positive things at home not done during non-pandemic everyday life and leaves time to go out and experience nature. These activities may bring about a sense of connection with someone or something and thus reduce loneliness (27).

This study thus investigates (a) the risk and resilience factors proposed above for loneliness in the general adult population during the strict social distancing NPIs implemented in Norway from March 12th 2020 against the COVID-19 pandemic and (b) the associations between loneliness and psychopathology symptoms. The implemented NPIs included not leaving home unless necessary, home isolation if infected, quarantine after exposure to possible infection, closure of kindergartens, schools, universities, and other public spaces, restrictions on traveling, and prohibitions of social gatherings and arrangements. At the first day of the data collection (March 31st), the total number of infected cases was 4,641 of a population of 5.4 millions and the number of new cases that day was 196. At the last day of the data collection (April 7th), the number of new cases was 221 (28). An investigation of the associations of loneliness with potential risk and resilience factors and other psychopathology provides a foundation for employing interventions that protect the general public against increased distress and dysfunction during the pandemic.

The following are the hypotheses (Hs) of the present study:

H1: Stable risk factors, such as lower age, lower educational level, not being married or in a civil union, not being in work and having a psychiatric diagnosis will be associated with more loneliness during the NPIs. The relationships between loneliness and gender, not having children, and being a refugee will be investigated exploratively.
H2: Of the state factors related to the pandemic and the NPIs, worry about jobs and/or the economy, worry about health (health anxiety), and worry and rumination in general will be associated with more loneliness, above and beyond the influence of the pre-existing stable risk factors.
H3: Doing new positive activities at home and experiencing nature will be associated with less loneliness when the influence of stable and state risk factors are controlled.
H4: Elevated loneliness will be associated with more depressive and anxiety symptoms.

Because variables related to loneliness may confound the relationship between loneliness and psychopathology symptoms, the variables showing significant associations for Hypotheses 1–3 will be controlled for. The presence of psychiatric diagnosis as an indicator of pre-NPI symptoms will be used as a covariate, irrespective of whether it is supported in Hypothesis 2.

## Materials and Methods

### Study Design and Participants

The design was a cross-sectional and epidemiological survey of the general adult Norwegian population during the COVID-19 pandemic. Eligible participants were all individuals aged 18 years and above who were living in Norway and thus experiencing identical NPIs, and who had provided informed consent to participate in the study. The period of data collection lasted seven days between March 31st 2020 and April 7th 2020. The NPIs were implemented from March 12th 2020 and held constant during the 2 weeks prior to data collection and during the data collection week. Furthermore, no new information was given by the government during this period with regard to changes in the NPIs, keeping expectation effects constant. The number completing the survey was 10,084. When re-contacted for further measurement, 23 out of these 10,084 participants requested to be removed from the study and not contacted further. In accordance with the General Data Protection Regulation law in Norway, use of the data of these 23 participants is precluded. Consequently, the overall sample size was updated from 10,084 to 10,061 participants.

The study was conducted in accordance with the guidelines of the Strengthening the Reporting of Observational Studies in Epidemiology statement (29). Ethical approval of the study was granted by the Regional Committee for Medical and Health Research and the Norwegian Centre for Research Data (reference numbers: 125510 and 802810, respectively) prior to data collection. The pre-registered protocol of this study can be found at Clinicaltrials.gov (Identifier: NCT04365881). Because of the sudden onset of the pandemic and the need for immediate observation of mental health reactions, we were unable to pre-register prior to data collection. Thus, we had access to the data at the date of registration, though no analysis was conducted related to the research plan. All elements of the submitted study adhere to the pre-registered protocol. The study is part of the Norwegian COVID-19 Mental Health and Adherence Project (30).

### Procedures

The survey was disseminated online in a systematic manner to give the adult population an equal opportunity to participate in the study. The dissemination procedure involved information about the survey through broadcasting on national, regional and local news channels and the provision of the online survey to a random selection of Norwegian adults on Facebook. The procedure is described in detail elsewhere (30).

The stopping rule for data collection was designed to ensure that the NPIs were held constant for 2 weeks prior to and the week during the data collection period, as well as controlling for expectation effects by stopping the data collection instantly once information concerning forthcoming modifications to the NPIs was given.

### Measures

The participants were asked to report the demographic variables of sex (male, female, transgender, intersex), identification with biological sex, age, education (not completed junior school, completed junior school, completed high school, currently studying, completed university degree), being a refugee, civil status (married, living in a civil union, single), number of children, employment status and presence of psychiatric diagnosis. Questions about suspecting being infected and about time staying home and reasons for this were included.

The UCLA Loneliness Scale-8 (ULS-8) (31) measures the frequency and intensity of aspects of loneliness, using a 1 (*never*), 2 (*rarely*), 3 (*sometimes*) and 4 (*often*) scale. A composite score was computed by summing the items after reverse coding when appropriate, with composites ranging from 8 to 32. The ULS-8 has demonstrated good psychometric properties (31, 32) and had a satisfactory internal consistency–Cronbach's α of 0.82–in the present sample.

The Patient Health Questionnaire-9 (33) consists of nine items covering the DSM-IV criteria for major depression scored on a four-point scale ranging from 0 (*not at all*) to 3 (*almost every day*) with total scores ranging from 0 to 27. The PHQ-9 has revealed good psychometric properties (33) and had a Cronbach's α of 0.88 in the present sample.

The Generalized Anxiety Disorder-7 (GAD-7) (34) consists of seven items covering the DSM-IV criteria for GAD scored on a four-point scale ranging from 0 (*not at all*) to 3 (*almost every day*) with total scores ranging from 0 to 21. The GAD-7 has revealed construct validity and reliability (34) and had an α of 0.88 in the present sample.

Symptoms of health anxiety were measured with two items from the validated Health Anxiety Inventory (HAI) (35)—Item 1: “I constantly have images of myself being ill” and Item 6: “I spend much of my time worrying about my health”–as well as an item measuring specific fear of being infected with coronavirus and an item measuring fear of dying from the coronavirus. These items are rated on a scale ranging from 0 (*never*) to 3 (*almost every day*) asking about symptomatology for the last 2 weeks. The sum score of these items had an α of 0.79 in the present sample.

Current worry about job and economy was measured by the items: “I am worried that I will lose my job” and “I am worried about my economy,” using a scale ranging from 0 (*never*) to 3 (*almost every day*). A general worry and rumination item was taken from the Cognitive Attentional Syndrome-1 (CAS-1) (36, 37): “How much time in the last week have you found yourself dwelling on or worrying about your problems? (0–8 Likert-type scale). Two coping behaviors were assessed: time being engaged in positive activities one would otherwise not have the time for during the non-pandemic everyday life and time spent experiencing nature.

### Statistical Analyses

The statistical analyses were conducted in the SPSS program version 25.0 (38). Hierarchical regression analyses were conducted using loneliness as the dependent variable. The first step examined the stable factors: age, gender, civil status, employment status, being a refugee, having children, and having a psychiatric diagnosis (H1). The second step added the NPI-related state variables worry about job and/or economy, health anxiety, and rumination and worry in general (H2). The final step incorporated the coping behaviors of doing new things and experiencing nature (H3).

Two separate hierarchical linear regression analyses were conducted using depressive symptoms (PHQ-9) and anxiety symptoms (GAD-7) as dependent variables (H4). In both analyses, the variables significantly predicting loneliness in the analyses of H1, H2, and H3 were included to control for potentially confounding factors. The presence of psychiatric diagnosis was also included as an indicator of pre-NPIs symptoms.

In all regression analyses, multicollinearity and other assumptions were checked, particularly if the multicollinearity assumption was violated (if VIF > 3 and tolerance <0.2) (39). Given the large sample size in this study, we chose the conservative level of p < 0.001 to determine significance. Effect sizes were estimated using part (semi-partial) correlations. A part correlation gives a less biased and more interpretable estimate of the strength of a predictive relationship than the standardized regression coefficients and the partial correlation coefficients (40). A part correlation is the correlation between the outcome and the aspects of the predictor unique from all the other predictors. As a type of correlation, its size can be evaluated according to Cohen's (41). criteria: small ≥ 0.10, medium ≥ 0.30, and large ≥ 0.50. A simple correlation size of 0.30 is usually used as the threshold of clinical relevance (42). However, as part correlations were used here, for which many potential confounders were controlled, we used the more lenient level of 0.20 for clinical relevance. There were no data missing in our set because the online survey system comprised only mandatory fields of response.

## Results

### Characteristics of Participants and Proportional Representation

National NPIs were employed in Norway from March 12, 2020, and the data collection in this study was conducted between March 31, 2020 and April 7, 2020. Consequently, at the time of measurement, the duration for which the respondents experienced NPIs ranged from 19 to 26 days. The 10,061 participants were aged 18–86 years, giving a mean age of 36.0 years (*SD* = 13.5). Table 1 reports the distribution of participants across age groups and other categorical variables. In terms of a proportional representation of the Norwegian population, more females (7,851, 78.0%) than males (2,184, 21.7%) responded. The sample was also not representative in terms of educational level, as 5644 (56.1%) had completed a university degree, compared to about 34.1% of the population (43). The proportion having a psychiatric diagnosis was 1,721 (17.1%) of 10,061, which reflects the lower end of the known 1-year prevalence of 16.7% to 25.0% in the adult population of Norway (44). The sample was further geographically representative of Norway, with the ratio of individuals from each region being reasonably proportionate to the population parameter. The percentage of people living in Eastern Norway is 58.3%, compared to 63.0% of the respondents who lived there (43). The corresponding numbers were 20.3 and 24.9% for Western Norway, 16.0 and 10.5% for Middle Norway, and 5.5 and 3.6% for Northern Norway.

**Table 1:** Levels of loneliness (ULS-8) during a 2-week period under non-pharmacological interventions (NPIs) related to the COVID-19 pandemic.

**Variables and categories**	***N***	**Mean (*SD*)**	**Test**
All participants	10,061	17.51 (4·90)	
**Sex**			
Female	7,851	17.73 (4.82)	
Male	2,184	16.70 (5.09)	
Transgender	22	19.91 (4.75)	
Intersex	4	15.00 (4.24)	*F*^a^ = 40.52, *p* < 0.001
**Identification with sex**			
No	51	19.80 (4.52)	
Yes	10,010	17.50 (4.90)	*t* = 3.35, *p* < 0.001
**Age groups**			
18–30	4,706	17.75 (4.75)	
31–44	2,849	17.75 (4.93)	
45–64	2,142	16.93 (5.10)	
65+	364	15.93 (4.85)	*F* = 28.88, *p* < 0.001
**Educational level**			
High school or lower	4,417	18.05 (5.00)	
University degree	5,644	17.08 (4.78)	*t* = 9.90, *p* < 0.001
**Partnership status**			
Unmarried or not in a civil union	5,310	18.36 (4.94)	
Married or in a civil union	4,751	16.56 (4.68)	*t* = 18.71, *p* < 0.001
**Employment status**			
Currently unemployed	1,921	19.07 (5.30)	
Currently employed	8,140	17.14 (4.73)	*t* = 15.67, *p* < 0.001
**Refugee status**			
Refugee	574	17.64 (4.98)	
Not refugee	9,487	17.50 (4.90)	*t* = 0.52, *ns*
**Children**			
Not having children	5,808	17.86 (4.83)	
Having children	4,253	17.04 (4.96)	*t* = 8.34, *p* < 0.001
**Psychological diagnosis**			
Having a diagnosis	1,721	20.77 (4.97)	
Not having a diagnosis	8,340	16.84 (4.61)	*t* = 31.75, *p* < 0.001

*ULS-8, UCLA Loneliness Scale-8*.

a *Intersex participants not included in the analysis because of few in number*.

Of the 10,061 participants, 3,583 (35.6%) reported suspecting being infected by COVID-19 during the 2-week period, 3,399 (33.8%) reported worries about losing their job and 5,920 (58.8%) reported worries about their personal economy. The majority (*n* = 7,952, 79.0%) of the participants had stayed home most of the days (at least 10) of the last two weeks, 1,429 (14.2%) had been in home isolation or quarantine because of potential or proved infection, 693 (6.9%) had stayed home because of the closure of their enterprise and 854 (8.5%) had been assigned to a home office by their employer.

### Levels of Loneliness Across Demographic Subgroups

Table 1 provides descriptive statistics of loneliness across different subgroups and presents the results of the statistical tests, which indicate that female participants were more lonely than male and transgender participants and that young (18–30 years) and young to middle aged (31–44 years) participants were more lonely than middle aged to old (45–64 years) and old (65+) participants. Those who did not identify with their biological sex had higher loneliness scores than those who did, those with an educational level of high school or lower had higher loneliness scores than those with a university degree, those living alone had higher scores than those who were married or in a civil union, unemployed attained higher loneliness scores than employed, and those not having children scored higher than those with children. Finally, those with a psychiatric diagnosis had markedly higher loneliness scores than those without a diagnosis.

### Correlates of Loneliness

Table 2 presents the results of the multiple regression analyses for loneliness as the dependent variable. In the first step that examined the stable variables, the regression model accounted for 13% of the variance in loneliness, adjusted *R*^2^ = 0.13. Being male was a significant correlate of loneliness, with men having lower levels of loneliness than women and than transgender and intersex counted together. Being transgender or intersex did not differ from males and females counted together. Increased age and higher education were associated with decreased loneliness. Being employed and being married or in a civil union were both associated with lower loneliness. Being a refugee and not having children were unrelated to loneliness. Having a psychiatric diagnosis was associated with more loneliness. Of these stable factors, civil status and psychiatric diagnosis attained a clinically significant effect of small size (part *r* ≥ 0.10). The other effects were negligible.

**Table 2:** Results of multiple regression with loneliness (ULS-8) as the dependent variable.

**Variables**	***B***	***SE***	**ß**	***t***	**Part *r***	***R^**2**^***	***ΔR*^2^**
**Step 1. Stable risk factors**						0.13	0.13^*^
Male	−0.66	0.11	−0.06	−5.79^*^	−0.05		
Transgender or intersex	−0.67	0.90	−0.01	−0.75	−0.01		
Age	−0.03	0.01	−0.08	−5.68^*^	−0.05		
Educational level	−0.15	0.10	−0.02	−1.50	−0.01		
Married or in a civil union	−1.60	0.11	−0.16	−15.25^*^	−0.14		
Employed	−1.36	0.13	−0.11	−10.61^*^	−0.10		
Refugee	0.29	0.20	0.01	1.48	0.01		
Having children	0.33	0.13	0.03	2.55	0.02		
Psychological diagnosis	3.37	0.13	0.26	26.46^*^	0.25		
**Step 2. State risk factors**						0.30	0.17^*^
Worry about job and economy	0.28	0.03	0.10	10.95^*^	0.09		
Health anxiety	0.10	0.02	0.05	5.07^*^	0.04		
General rumination and worry	0.91	0.02	0.38	37.15^*^	0.31		
**Step 3. Coping behaviors**		07				0.32	0.02^*^
Doing new things at home	−0.66	0.05	−0.12	−14.07^*^	−0.12		
Experience nature	−0.23	0.04	−0.05	−5.23^*^	−0.04		

*ULS-8, UCLA Loneliness Scale-8*.

* *p < 0.001*.

In the second step, which added state variables, the model explained 30% of the variance in loneliness, adjusted *R*^2^ = 0.30. More worry about job and economy, more health anxiety and more general rumination and worry were all related to more loneliness. General rumination and worry achieved a clinically significant effect of medium size (part *r* > 0.30), whereas the effects of the other state variables were negligible.

In the third step, which included coping behaviors, the model explained 32% of the variance in loneliness, adjusted *R*^2^ = 0.32. Doing positive activities at home not done otherwise and experiencing nature were both related to less loneliness. Only doing new activities achieved an effect of small size (part *r* ≥ 0.10).

### Loneliness as a Correlate of Depression and Anxiety

Tables 3, 4 present the results of multiple regression analyses using depression and anxiety, respectively, as dependent variables. Loneliness and all variables that were significantly related to loneliness were used as independent variables. Loneliness was related to depression (part *r* = 0.19) and anxiety (part *r* = 0.10).

**Table 3:** Results of multiple regression with depression (PHQ-9) as the dependent variable.

**Variables**	***B***	***SE***	**ß**	***t***	**Part *r***	***R^**2**^***	***ΔR^**2**^***
						0.62	0.62^*^
Male	−0.64	0.09	−0.05	−7.27^*^	−0.05		
Age	−0.06	0.00	−0.13	−18.21^*^	−0.11		
Married or in a civil union	−0.43	0.08	−0.04	−5.73^*^	−0.04		
Employed	−0.58	0.10	−0.04	−5.88^*^	−0.04		
Psychological diagnosis	2.39	0.10	0.16	22.92^*^	0.14		
Worry about job and economy	0.26	0.02	0.08	11.57^*^	0.07		
Health anxiety	0.16	0.02	0.07	9.42^*^	0.06		
General rumination and worry	1.14	0.02	0.42	50.88^*^	0.31		
Doing new things at home	−0.52	0.04	−0.08	−12.71^*^	−0.08		
Experience nature	−0.23	0.04	−0.04	−6.12^*^	−0.04		
Loneliness	0.26	0.01	0.23	30.43^*^	0.19		

*PHQ-9, Patient Health Questionnaire-9*.

* *p < 0.001*.

**Table 4:** Results of multiple regression with anxiety (GAD-7) as the dependent variable.

**Variables**	***B***	***SE***	**ß**	***t***	**Part *r***	***R^**2**^***	***ΔR^**2**^***
						0.65	0.65^*^
Male	−0.44	0.07	−0.04	−7.27^*^	−0.04		
Age	−0.02	0.00	−0.07	−6.42^*^	−0.06		
Married or in a civil union	0.24	0.06	0.03	4.12^*^	−0.02		
Employed	−0.80	0.08	−0.01	−1.03	−0.01		
Psychological diagnosis	1.38	0.08	0.11	16.94^*^	0.10		
Worry about job and economy	0.17	0.02	0.06	9.70^*^	0.06		
Health anxiety	0.42	0.01	0.21	31.93^*^	0.19		
General rumination and worry	1.17	0.02	0.52	66.68^*^	0.40		
Doing new things at home	−0.08	0.03	−0.02	−2.42^*^	−0.01		
Experience nature	0.13	0.03	0.03	4.31^*^	0.03		
Loneliness	0.12	0.01	0.12	17.23^*^	0.10		

*GAD-7, Generalized Anxiety Disorder-7*.

* *p < 0.001*.

### Sensitivity Analyses

Sensitivity analyses were conducted after selecting a random sample of participants to match the number of males and females as well as the proportion of education levels to correctly reflect the Norwegian adult population. These analyses, which involved 3,098 of the participants, revealed identical results for the group comparisons and the regression analyses in terms of significant findings and level of effect sizes. Thus, the robustness of the presented results was supported.

## Discussion

The present study investigated the mental health problem of loneliness in an adult Norwegian community sample (*N* = 10,061) during a period involving the globally in-practice NPIs used to impede transmission of the COVID-19 virus. The aim was to investigate (a) the potential risk and resilience factors for loneliness in the Norwegian population during the strict social distancing NPIs and (b) the associations between loneliness and psychopathology symptoms.

The results showed that nearly 80% had stayed home most of the time, indicating that the government-initiated social distancing measures had been adhered to. Thus, a large proportion of individuals had abstained from their usual social life, with some examples including informal contact with colleagues at work, general interaction with peers outside one's household, visits of grandchildren to grandparents, organized sport activities for young adults and physical gatherings with friends and family. Although digital and phone communication could have replaced some of the physical interactions, the extensive restrictions on social interaction are likely to have been accompanied by increased loneliness.

Comparisons across demographic groups led to the identification of the following subgroups with increased loneliness: females, people who do not identify with their own biological sex, young and young to middle-aged people, those with a lower educational level, singles, unemployed, those who do not have children and those who have a psychiatric diagnosis.

In regression analyses, the possible correlations between the independent variables are accounted for, and only the unique contribution of each of these variables to the dependent variable is assessed. Thus, the results may differ somewhat from the group comparisons presented above. Among the stable factors and as hypothesized, being younger, single, less educated, unemployed and having a psychiatric diagnosis were all related to increased levels of loneliness. Exploratively, being male was associated with less loneliness, whereas not having a child and being a refugee were unrelated to loneliness. Part correlations indicated that being single and having a diagnosis achieved small effect sizes. The size of the other relationships was negligible.

More loneliness among younger people was also found in a longitudinal study of adults in the United States before and during the pandemic (45) and in a longitudinal study of adults in the United Kingdom during the pandemic (46). Young people probably need more social contact and thus may suffer more during the increased isolation. As with the other findings of this study, we do not know whether the younger adults had increased loneliness also before the pandemic.

Among single individuals, comparison processes may strengthen feelings of loneliness, in addition to the direct effect of social distancing. These individuals may withdraw to aloneness and may feel lonelier when comparing their situation with the situations of those who withdraw to their core family.

Having a psychiatric diagnosis is known to be associated with loneliness, as loneliness may be both a cause and an effect of mental disorders, and loneliness and mental disorders may be common effects of life events (11). Moreover, having a diagnosis may lead people to feel different and isolated (47).

Among the state risk factors and as hypothesized, more rumination and worry in general was associated with stronger loneliness, showing a notable medium effect size. This result is consistent with the proposal that rumination and worry may lead to loneliness. The mechanisms tend to be a strain in close relationships, promote an inward focus leading to less engagement in other people and outward tasks, and encourage behavioral inactivity that hinders access to corrective experiences (21–23). Future studies should assess whether rumination and worry create loneliness, whether loneliness creates rumination and worry, or whether the relationship is reciprocal.

As hypothesized, more worry about job and economy was associated with more loneliness, showing a small effect size. The work-related and economic consequences of the pandemic and the implemented NPIs involve a lot of job and financial insecurity, especially for people on low incomes. Thus, as many as 58.8% of the sample worried about their economy for at least some of the days during the last 2 weeks. The results of this study suggest that worry about job and economy has some influence on loneliness. More health anxiety was also related to more loneliness, but the size of this relationship was negligible.

Among the two coping behaviors hypothesized to be associated with loneliness, doing new things at home not done otherwise was negatively related to loneliness with a small effect size. Experiencing nature was also negatively related to loneliness, but the size of this relationship was negligible.

Symptoms of depression and anxiety were prevalent in the present sample. As reported elsewhere (30), 30.8% met the diagnostic cut-off for depression and 25.6% met the cut-off for GAD. Loneliness predicted both depressive and anxiety symptoms with small effect sizes when all potential confounders and psychiatric diagnosis were controlled for. The relationship was more marked for depression, suggesting that loneliness is more closely related to depression than anxiety. Thus, loneliness may be a potential risk factor for depression, and both loneliness and depression may involve internal feedback processes leading to persistence (11) even after the pandemic is controlled and the NPIs lifted.

The strengths of this study are that it captured the effects of NPIs momentarily as they happened and were held constant during the measurement period. Thus, this study provides the grounds for evaluation and modification of these strategies in real time, as they are still in practice worldwide. A limitation of this study is that random sampling was not conducted because of the urgency of the data collection. Thus, those who chose to respond may have specific features that may affect the results. However, effort was taken to give the adult population an equal opportunity to participate, and the resulting sample turned out to be relatively representative of the adult Norwegian population in terms of the proportion of sub-groups. Moreover, the large sample size allowed us to control for biases in gender and education level through *post-hoc* stratification and sensitivity analyses. These analyses yielded almost identical results to the main analyses, which supports the robustness of the presented results. A further limitation is the cross-sectional design, which impairs the ability to draw conclusions about temporal precedence and causal direction. From our data, it is also impossible to know to the extent to which the obtained relationships were present before the COVID-19 and to what extent they were accentuated during the pandemic. Owing to the lack of ULS-8 data for the Norwegian population in non-pandemic circumstances, we could not provide evidence that the level of loneliness reflected a pandemic increase. Additional limitations are that the variables were assessed by self-report, that the measures of health anxiety and worry about job and economy were self-constructed and are unvalidated, and that rumination and worry were measured by only one item, making this variable prone to measurement error.

Although the causal status of the identified correlates of loneliness is uncertain, two of the obtained part correlations had a clinically relevant size (≥0.20) and tentatively suggest some targets of intervention. For people with a psychological diagnosis who attend mental health services, the disruption of these services caused by the implementation of NPIs is likely to increase social isolation and loneliness. As a substitute, remotely delivered methods should be used to provide connectivity and support to patients. Given the potential influence of rumination and worry and the existence of evidence-based psychological therapies for these processes (48) psychological first-aid self-help programs and low-threshold internet-based therapies should be established.

In conclusion, the present survey suggests that people adhere to government-initiated social distancing NPIs during pandemics and withdraw to their homes. It is therefore likely that loneliness increases, although this could not be demonstrated in this study due to a lack of adequate comparison data. Given the strongly increased morbidity and mortality associated with social isolation and loneliness (8, 49), this is a serious downside of the NPIs. The results of the survey further suggests that single individuals and those with a psychiatric diagnosis are especially vulnerable, that loneliness is closely associated with rumination and worry, that doing new positive things at home may mitigate loneliness and that loneliness is associated with depression and anxiety. Longitudinal studies extending through and beyond pandemics are necessary to examine the extent to which increased loneliness persists after the social distancing measures are lifted, whether loneliness leads to symptoms or *vice versa* and the possible mediating relationships between rumination and worry, loneliness and symptoms.

## Data Availability Statement

The present dataset includes information about specific behaviors, trait variables, and health outcomes of a large and representative sample of the Norwegian population. In accordance with the guidelines of The Norwegian Centre for Research Data (NSD) and The Regional Committee for Medical and Health Research Ethics (REK), access to the data may be provided to qualified investigators whose proposed use of the data have been approved by all relevant independent review committees in Norway, including REK and NSD, and whose research plans provide a defensible proposal and methodologically sound design with the aims approved by REK, NSD, the university ethics board, and other necessary organizations that require an application process for providing access to sensitive data containing predictive information on a large and representative sample of the Norwegian population. Requests to access the datasets should be directed to Sverre Urnes Johnson, s.u.johnson@psykologi.uio.no.

## Ethics Statement

The studies involving human participants were reviewed and approved by The Regional Committee for Medical and Mental Health Research Ethics, Gullhaugveien 1-3, 0484 Oslo, Norway. The patients/participants provided their written informed consent to participate in this study.

## Author Contributions

AH analyzed the data, wrote the first draft of the paper, and revised it based on the SJ's and OE's comments. OE administered the collection of the data and conducted data curation. All authors contributed to the conceptualization and methodology of the study.

## Conflict of Interest

The authors declare that the research was conducted in the absence of any commercial or financial relationships that could be construed as a potential conflict of interest.

## Abbreviations

COVID-19: coronavirus disease 2019
NPIs: non-pharmacological interventions
H: hypothesis
ULS-8: UCLA Loneliness Scale-8
PHQ-9: Patient Health Questionnaire-9
GAD-7: Generalized Anxiety Disorder-7
HAI: Health Anxiety Inventory
CAS-1: Cognitive Attentional Syndrome-1
M: mean
SD: standard deviation
N: number
B: regression coefficient
SE: standard error
ß: standardized regression coefficient
R^2^: coefficient of determination
r: Pearson's correlation
OECD: Organization of Economic Co-operation and Development.
